# Irrational Use of Selected Herbal Medicines During Pregnancy: A Pharmacoepidemiological Evidence From Yemen

**DOI:** 10.3389/fphar.2022.926449

**Published:** 2022-07-19

**Authors:** Mansoor Ahmed, Jung Hye Hwang, Mohammed Nasr Ali, Shafekah Al-Ahnoumy, Dongwoon Han

**Affiliations:** ^1^ School of Public Health, Dow University of Health Sciences, Karachi, Pakistan; ^2^ Department of Preventive Medicine, Hanyang University College of Medicine, Seoul, South Korea; ^3^ Department of Global Health and Development, Graduate School, Hanyang University, Seoul, South Korea; ^4^ Institute of Health Services Management, Hanyang University, Seoul, South Korea; ^5^ Department of Obstetrics and Gynecology, Hanyang University College of Medicine, Seoul, South Korea; ^6^ Ministry of Public Health & Population Department of Quality and Patient Safety, Sana’a, Yemen; ^7^ Al-Kuwait University Hospital, Sana’a, Yemen

**Keywords:** pharmacoepidemiology, rational use, pregnancy outcome, Yemen, herbal medicine (HM)

## Abstract

**Background:** Recent research indicates irrational use of herbal medicine (HM) during pregnancy that can be harmful to the mother and development of baby. However, no study has been conducted to explore the use of HM among pregnant women in a conflict region.

**Methods:** This was a cross-sectional research conducted in July and August in the year 2017, at three hospitals in Sana’a, Yemen. Postpartum women were interviewed via the structured instrument to collect data on patterns of HM used during pregnancy.

**Results:** A total of three hundred postpartum women participated in the study, with 59.3% (178) of them using at least one modality of HM in the previous pregnancy. Cinnamon (*Cinnamomum verum* L), ginger (*Zingiber officinale* Z), barley (*Hordeum vulgare* P), and garlic (*Allium sativum* A) were the most commonly used HM. Statistical analysis showed that consumers of HM had a higher rate of caesarian section and complications during and after the delivery.

**Conclusion:** Consumption of HM during pregnancy was high including contraindicated modalities such as cinnamon (*C. verum* L) and barley (*H. vulgare* P). The use of potentially harmful modalities and association with complications pose a threat to the well-being of mothers and newborns. Further studies in this area will be helpful to generate evidence for the rational use of HM during pregnancy.

## Introduction

The fierce armed conflict that intensified more than sixyears ago has made Yemen undergo the world’s largest humanitarian crisis. More than 20 million people need health assistance, and the number of those in acute need exceeds 11 million (UN Country Team in Yemen and UN Office for the Coordination of Humanitarian Affairs, 2017). According to the World Health Organization (WHO), only about a quarter of the Yemeni population has access to healthcare services (Al-Adhroey et al., 2020) and the health system is close to collapse (WHO, 2015). The maternal mortality ratio in Yemen is one of the highest in the world, i.e., 164/100,000 live births (Butt et al., 2022). It is one of the high-alert countries for maternal mortality in the Fragile Countries Index (UN Country Team in Yemen and UN Office for the Coordination of Humanitarian Affairs, 2017). Shortage of specialized staff, particularly lack of female doctors, poor coverage, and dearth of medical supplies influence maternal health-seeking behavior (UN Country Team in Yemen and UN Office for the Coordination of Humanitarian Affairs, 2017). One such influence is the escalated use of complementary and alternative medicine (CAM) in Yemen (Al-Adhroey et al., 2020). The traditional medicine (or CAM) in Yemen belongs to the old Arabic medicine and has its foundations in Greek medicine (Borg, 2017).

One of the widely used CAM during pregnancy is the consumption of herbal medicine (HM). In general, HM is considered comparatively more affordable, accessible, and considered a safe alternative to modern medicine. The prevalence of using HM during pregnancy is indicated in developed countries (Heitmann et al., 2015) as well as developing countries (Jaradat and Adawi, 2013; Hwang et al., 2016; Ahmed et al., 2018). In these countries HM during pregnancy is most commonly used to relieve nausea/vomiting, constipation, and the common cold, and to improve health status that may facilitate a normal delivery, the birth of a healthy baby, and supply of breast milk. The popularity of HM during pregnancy is because of the fact that several conventional medications are contraindicated in pregnancy because of the possible harm to mother and fetus. HM being natural are believed to be safe to use even during pregnancy. However, in most of the developing countries, HM can be easily obtained without a prescription, and plants with medicinal properties are usually purchased as unregulated food products that do not go through standard pharmaceutical regulatory processes (Sachan et al., 2016). Therefore, there remains a risk of adulteration with undeclared elements and contamination with other products such as arsenic (Liu et al., 2018). Moreover, as herbs comprise active biological substances that have the potential to mediate pharmacological actions, HM use in pregnancy may produce adverse effects (Ahmed et al., 2017).

Due to the paucity of epidemiological studies on the rational use of HM in pregnancy, pregnant women have little knowledge in this regard. A study reported that 39% of pregnant women had used modalities of HM that were either possibly harmful to use in pregnancy or evidence on the safety was not available (Nordeng and Havnen, 2004). Recently, a multinational study, using current scientific literature, classified the safety of 126 different modalities of HM used by pregnant women. The study emphasized that, in pregnancy, only 22% of these modalities were safe (Kennedy et al., 2016). In a more recent review of HM use by Asian pregnant women, we reported that out of 31 most commonly used modalities, 18 were potentially unsafe and thus irrational to use in pregnancy (Ahmed et al., 2017).

Studies have also been conducted to explore the possible negative effects of HM on the outcome of pregnancy. A cohort study from Taiwan highlighted that use of some Chinese HM in pregnancy was related to a higher incidence of congenital malformation of multiple organs (Chuang et al., 2006). More recently, another study reported that consumption of Licorice (*Glycyrrhiza glabra* F) and Chamomile (*Matricaria recutita* A) during pregnancy was associated with an increased incidence of preterm labor and threatening miscarriage, and smaller size of neonates (Cuzzolin et al., 2010). It is also noteworthy that in several countries health professionals and the general public can report adverse effects of medications along with HM via a structured form. This is commonly called Spontaneous Reporting (Coleman and Pontefract, 2016). However, little is known regarding the herbal medication errors and adverse drug reactions in Yemen (Alshakka et al., 2019). As a result, the adverse effects of medicines, including HM, experienced by the public may remain unreported.

This indicates the need for pharmacoepidemiological studies, particularly among less studied groups such as pregnant women. One study from Yemen highlighted the risks of using HM during pregnancy and lactation. However, it was only based on the perception of community pharmacists and technicians. The study highlighted incorrect perceptions about the risks involved (Thabit et al., 2020). Another study from Palestine reported differences between traditional and scientific uses of HM during pregnancy and lactation (Eid and Jaradat, 2020). A cohort study from Iran did not find any association between newborn outcomes and maternal consumption of HM during pregnancy (Raoufinejad et al., 2020). The choice of HM use is a culture-specific phenomenon; thus it is essential to conduct country-specific studies. To the best of our understanding, no such study is reported from Yemen. This research aimed to determine the prevalence and characteristics of HM use by pregnant women in Yemen and to understand its rationality of it.

## Materials and Methods

### Study Design

This was a descriptive cross-sectional study conducted at three hospitals in Sana’a, Yemen—Al-70 Hospital, Al-Gumhouri Teaching Hospital, and Safe Motherhood Specialized Hospital.

### Study Setting and Participants

The inclusion criterion was postpartum women before discharge from the hospitals to minimize the recall bias. Whereas women who were being managed for severe conditions such as in the intensive care unit were excluded. Those who were mentally disabled or unable to speak were also excluded.

### Study Size

The sample size for this study was calculated based on the formula using the confidence interval of proportion: $n=z_{\alpha /2}^{2}\cdot \frac{pq}{d^{2}}$ . Moreover, as there were no studies on the use of HM among pregnant women in Yemen, studies from Arab countries were used as references (Amasha and Jarrah, 2012; Jaradat and Adawi, 2013; Hwang et al., 2016). It gave the minimum sample size of 284. However, 400 participants were invited to join in order to account for a possible non-response.

### Data Collection

A total of six individuals participated in the data collection. Two supervisors (MNA & SA) monitored the entire process of data collection. The supervisors were in regular contact with the principal investigator (MA). Each data collector captured data from different participants. In order to ensure quality data collection and to avoid bias among different data collectors, there was comprehensive training at the start and in the mid of the data collection. Moreover, experienced female data collectors were recruited to ensure accuracy. Experienced female data collectors are more sociable and generate a good response rate (Sinibaldi et al., 2009). A total of 400 women were asked to participate in the survey when they were moved to maternity wards after the delivery. The participation was voluntary and confidentiality was guaranteed. Data related to labor, post-labor, and newborn baby were collected from hospital records, whereas the rest of the questions were asked by the participants themselves. The survey was conducted between July and August 2017.

### Survey Instrument

The first draft of the survey instrument was developed based on existing literature and our observations of the target population. As this study measured several health indicators using diverse questions, the statistical validity and reliability were not applicable for the items that measured different constructs (Streiner, 2003). Face validity of the instrument was conducted by two doctors in the target hospitals and a practitioner of traditional medicine. Moreover, the content validity of the instrument was carried out with the help of physicians working in the obstetrics department in the target hospitals. The instrument was first developed in English and then translated into the Arabic language for the target population. The Arabic questionnaire was back-translated into English for accuracy. Then a pilot study was conducted on a sample of 20 women. Based on the results, it was revised again. The final version of the questionnaire included 32 items. It was divided into five sections. Most of the question items were based on nominal scale data with close-ended questions, and mostly had an additional open-ended option of “Others.”

The first section comprised generic questions such as exposure to secondary smoking, health status, gravidity, mode of delivery, use of *Khat* (*Catha edulis* C), and antenatal care services. The second section consisted of questions on prenatal complications, during labor, and postnatal maternal complications. The third section comprised items on the use of HM such as types of HM along with their indications, any side effects, recommending source, reason for using and not using, and disclosure of use to doctor or midwife. The fourth section of the instrument is comprised of items related to the characteristics of the newborn. The final section included items on the sociodemographic profile of the women. The ongoing conflict in Yemen has affected the functionality of government institutions and the socioeconomic status of the country in general. As a consequence, salaries are not paid to employees on regular basis, especially those in the public sector (UN Country Team in Yemen and UN Office for the Coordination of Humanitarian Affairs, 2017). Therefore, instead of monthly household income, we measured household economic status, using two question items: ownership of a car and type of electricity in the household. For details, please see the data collection tool attached as a Supplementary File.

### Statistical Analysis

The data were analyzed using Statistical Package for Social Sciences (SPSS) v. 21. Respondents were classified as HM users when they used a minimum of one modality of HM during their previous pregnancy, while others were categorized as non-users. The frequency and percentage were calculated in descriptive statistics. Pearson’s chi-square and Fisher’s exact tests were utilized to find out the correlation for characteristics between users and non-users of HM. The resultant significant associations were further analyzed using multiple logistic regression. A *p*-value less than 0.05 indicated a statistically significant difference in all analyses.

### Ethical Clearance

The ethical approval was granted by the Institutional Review Board on Human Subjects Research and Ethics Committees, Hanyang University, Seoul, Korea (HYI-17-067-2). All participants gave written informed consent.

## Results

### Sociodemographic Characteristics

A total of 400 women were invited to participate in the study and only 300 agreed to do so, generating a response rate of 75%. Thus, data from 300 participants were used in the analysis. Sociodemographic profile of the participants is given in Table 1. Mean age of the respondents was 27.16 ± 6.49 years. Fifty-six percent aged 21–30 years, 75.3% belonged to urban areas, 50.7% had education up to elementary school, 93% were unemployed, 76.7% were from the middle economic group, and it took less than 30 min for 54% participants to reach the nearest health facility.

**TABLE 1:** Sociodemographic characteristics of participants.

Variables	Total = 300 N (%)	HM users = 178 N (%)	Non-users = 122 N (%)	*p* value
Mean age (years)	27.16 ± 6.49			
20 years and below	51 (17)	28 (15.7)	23 (18.9)	0.648
21–30 years	168 (56)	99 (55.6)	69 (56.6)	
31 years and above	81 (27)	51 (28.7)	30 (24.6)	
Residence				
Rural	74 (24.7)	29 (16.3)	45 (36.9)	**0.000** *X* ^*2*^ = 16.520
Urban	226 (75.3)	149 (83.7)	77 (63.1)	
Education level				
Elementary school or under	152 (50.7)	69 (38.8)	83 (68)	**0.000** *X* ^*2*^ = 24.809
High school or above	148 (49.3)	109 (61.2)	39 (32)	
Employed				
Yes	21 (7)	18 (10.1)	3 (2.5)	**0.011** *X* ^*2*^ = 6.513
No (housewife)	279 (93)	160 (89.9)	119 (97.5)	
Household economic status				
Low	26 (8.7)	13 (7.3)	13 (10.7)	0.109
Middle	230 (76.7)	133 (74.7)	97 (79.5)	
High	44 (14.7)	32 (18)	12 (9.8)	
Time to the nearest health facility				
Less than 30 min	162 (54)	79 (44.4)	83 (68)	**0.000** *X* ^*2*^ = 18.058
30 min to 1 h	94 (31.3)	71 (39.9)	23 (18.9)	
More than 1 h	44 (14.7)	28 (15.7)	16 (13.1)	

HM, herbal medicine.

### Medical Characteristics

Tables 2, 3 demonstrate medical characteristics. In total 68.7% of childbirths were done via caesarian section, 71.7% of the childbirths were full-term, 49.7% of women had four or more antenatal visits, 52% of the women were exposed to secondary smoking, 63.7% chewed *Khat* (*C. edulis* C) in pregnancy, 62.3% were multigravida, 40.3% women had their first pregnancy aged 19 years or under, 71.7% used HM prior to last pregnancy, and 53.3% experienced at least one morbidity during pregnancy.

**TABLE 2:** Medical characteristics of participants.

Variables	Total = 300 N (%)	HM users = 178 N (%)	Non-users = 122 N (%)	*p* value
Prior use of herbal medicine				
Yes	215 (71.7)	166 (93.3)	49 (40.2)	**0.000** *X* ^*2*^ = 100.495
No	85 (28.3)	12 (6.7)	73 (59.8)	
Antenatal visits				
None	26 (8.7)	7 (3.9)	19 (15.6)	**0.002** *X* ^*2*^ = 12.946
1 to 3 times	125 (41.7)	75 (42.1)	50 (41)	
4 or more times	149 (49.7)	96 (53.9)	53 (43.4)	
Exposed to passive smoking				
Yes	156 (52)	102 (57.3)	54 (44.3)	**0.026** *X* ^*2*^ = 4.932
No	144 (48)	76 (42.7)	68 (55.7)	
*Khat* (*Catha edulis* C) chewing in pregnancy				
Yes	191 (63.7)	122 (68.5)	69 (56.6)	**0.034** *X* ^*2*^ = 4.493
No	109 (36.3)	56 (31.5)	53 (43.4)	
Gravidity				
Primigravida	113 (37.7)	69 (38.8)	44 (36.1)	0.636
Multigravida	187 (62.3)	109 (61.2)	78 (63.9)	
Age at first pregnancy				
19 years or under	121 (40.3)	61 (34.3)	60 (49.2)	**0.013** *X* ^*2*^ = 8.727
20–24 years	114 (38)	70 (39.3)	44 (36.1)	
25 years or above	65 (21.7)	47 (26.4)	18 (14.8)	
Morbidities during pregnancy				
Yes	160 (53.3)	114 (64)	46 (37.7)	**0.000** *X* ^*2*^ = 20.178
No	140 (46.7)	64 (36)	76 (62.3)	

HM, herbal medicine.

**TABLE 3:** Logistic regression model showing factors predicting HM use during pregnancy.

Variables	Categories	*p* value	Odds ratio (OR)	Confidence interval for OR	
				Lower	Upper
Residence	Urban	**0.000**	4.587	2.206	9.539
	Rural (ref)				
Education (school)	Elementary or under (ref)	**0.003**	2.352	1.342	4.122
	High or above				
Travel time to nearest health facility	Less than 30 min (ref)	0.000	—	—	—
	30 min to 1 h	**0.000**	3.647	1.951	6.818
	More than 1 h	**0.001**	5.023	2.005	12.584
Prior use of herbal medicine	Yes	**0.000**	24.284	10.926	53.975
	No (ref)				
Morbidities during pregnancy	Yes	**0.000**	3.850	1.990	7.448
	No (ref)				

### Characteristics of Neonates

Profile of neonates is given in Table 3. For birth weight, 67.7% were between 2.5 and 3 kg. Symptoms were reported in only 18.3% of babies. Out of 300 babies, 49% were boys and 51% were girls (not shown in tables).

### Characteristics of Herbal Medicine Users

Out of 300 respondents (Tables 1, 2), 59.3% (178) used a minimum of one type of HM in their last pregnancy. HM, use was more frequent among women with age 21–30 years (55.6%), living in urban areas (83.7%), unemployed (89.9%), who had an education of high school or above (61.2%), belonged to the middle economic group (74.7%), and could get to the nearest health facility within 30 min (44.4%). Chi-square test showed that HM use was significantly associated with living in urban areas (*X* ^*2*^ = 16.520; *p* value < 0.05), higher level of education (*X* ^*2*^ = 24.809; *p* value < 0.05), unemployment (*X* ^*2*^ = 6.513; *p* value < 0.05), and short time to the nearest health facility (*X* ^*2*^ = 18.058; *p* value < 0.05).

Regarding medical characteristics, 93.3% of HM users had used HM before the pregnancy, had four or more antenatal care visits (53.9%), were exposed to passive smoking at home (57.3%), chewed *Khat* (*C. edulis* C) (68.5%), had first pregnancy between 20 and 24 years (39.3%), and were multigravida (61.2%). Chi-square test showed that earlier use of HM (*X* ^*2*^ = 100.495; *p* value < 0.05), age at first pregnancy (*X* ^*2*^ = 8.727; *p* value < 0.05), four or more antenatal visits (*X* ^*2*^ = 12.946; *p* value < 0.05), exposure to passive smoking (*X* ^*2*^ = 4.932; *p* value < 0.05), *Khat* (*C. edulis* C) chewing (*X* ^*2*^ = 4.493; *p* value < 0.05), and morbidities during pregnancy (*X* ^*2*^ = 20.178; *p* value < 0.05) were significantly associated with its use during pregnancy. The logistic regression model (Table 3) confirmed that HM use was predicted by urban residence, high school or above education, longer travel time to the nearest health facility, prior use of herbal medicine, and morbidities during pregnancy.

### Herbal Medicine Use and Pregnancy Outcome

Table 4 demonstrates the use of HM in terms of pregnancy outcomes. Chi-square analysis indicated that HM use during pregnancy was significantly related with perceived health status (*X* ^*2*^ = 4.336; *p* value < 0.05), caesarian section delivery (*X* ^*2*^ = 7.452; *p* value = 0.006), and complications during and after childbirth (*X* ^*2*^ = 20.755; *p* value = 0.000). Logistic regression model (Table 5) confirmed that HM use predicted good health status, cesarean section, and complications after childbirth.

**TABLE 4:** Pregnancy outcomes of participants.

Variables	Total = 300 N (%)	HM users = 178 N (%)	Non-users = 122 N (%)	*p* value
Perceived health status				
Good	193 (64.3)	123 (69.1)	70 (57.4)	**0.037** *X* ^*2*^ = 4.336
Fair or worse	107 (35.7)	55 (30.9)	52 (42.6)	
Type of last delivery				
Normal (inc. forceps)	94 (31.3)	45 (25.3)	49 (40.2)	**0.006** *X* ^*2*^ = 7.452
Caesarian	206 (68.7)	133 (74.7)	73 (59.8)	
Pregnancy term				
Full term	215 (71.7)	128 (71.9)	87 (71.3)	0.910
Pre term	85 (28.3)	50 (28.1)	35 (28.7)	
Complications during and after childbirth				
Yes	146 (48.7)	106 (59.6)	40 (32.8)	**0.000** *X* ^*2*^ = 20.755
No	154 (51.3)	72 (40.4)	82 (67.2)	
Birth weight				
Less than 2.5 kg	53 (17.7)	30 (16.9)	23 (18.9)	0.473
2.5–3 kg	203 (67.7)	125 (70.2)	78 (63.9)	
More than 3 kg	44 (14.7)	23 (12.9)	21 (17.2)	
Neonatal symptoms				
Yes	55 (18.3)	35 (19.7)	20 (16.4)	0.472
No	245 (81.7)	143 (80.3)	102 (83.6)	

HM, herbal medicine.

**TABLE 5:** Logistic regression model showing herbal medicine use predicting the outcome of pregnancy.

Outcome	*p* value	Odds ratio (OR)	Confidence interval for OR	
			Lower	Upper
Good health status	**0.034**	1.699	1.042	2.770
Caesarean section	**0.024**	1.796	1.082	2.981
Complications during and after childbirth	**0.000**	2.801	1.711	4.585

### Modalities of Herbal Medicines and Indications

Self-reported indications pertaining to each modality of HM are given in Table 6. The most frequently used HM—Cinnamon (*C. verum* L)—was used to facilitate delivery (92.8%). Ginger (*Z. officinale* Z) was most frequently used for cold/flu (40.5%), Barley (*H. vulgare* P) was used for urinary tract infection (81.1%), and Garlic (*A. sativum* A) was most frequently used for abdominal pain (50%).

**TABLE 6:** Reported indications of herbal medicine according to each modality (multiple choice).

Herbal modality	Reported indications	N (%)
Cinnamon (*Cinnamomum verum* L = 70)	Facilitate delivery	65 (92.8)
	Abdominal pain	5 (7.1)
	Fatigue	2 (2.8)
	Constipation	1 (1.4)
	Joint pain	1 (1.4)
	Reduce weight	1 (1.4)
Ginger (*Zingiber officinale* Z = 42)	Cold/flu	17 (40.5)
	Heartburn	14 (33.3)
	Nausea/vomiting	14 (33.3)
	Cough	4 (9.5)
Barley (*Hordeum vulgare p* = 37)	Urinary tract infection	30 (81.1)
	Facilitate delivery	6 (16.2)
	Heartburn	1 (2.7)
	Relaxant	1 (2.7)
	Nephritis	1 (2.7)
Garlic (*Allium sativum* A = 32)	Abdominal pain	16 (50)
	Hypertension	13 (40.6)
	Cold/flu	4 (12.5)
	Fatigue	2 (6.25)
Lemon tea (*Citrus limon* R = 25)	Cough	14 (56)
	Nausea/vomiting	9 (36)
	Flu	4 (16)
	Heartburn	2 (8)
	Constipation	1 (4)
Dates (*Phoenix dactylifera* A = 21)	Facilitate delivery	15 (71.4)
	Anemia	5 (23.8)
	Improve immunity	1 (4.8)
	Nausea/vomiting	1 (4.8)
Peppermint (*Mentha piperita* L = 16)	Heartburn	11 (68.75)
	Abdominal/gastric pain	6 (37.5)
	Cold/flu	2 (12.5)
Fenugreek (*Trigonella foenum-graecum* F = 16)	Facilitate delivery	15 (93.75)
	Gall bladder pain	1 (6.25)
	Indigestion	1 (6.25)
Roselle (*Hibiscus sabdariffa* M = 14)	Hypertension	8 (57.1)
	Urinary tract infection	3 (21.4)
	Cough	2 (14.3)
	Menstrual disturbances	1 (7.1)
	Fatigue	1 (7.1)

### Side Effects

The most common self-reported side effects after HM use during pregnancy were nausea/vomiting (6), diarrhea (4), abdominal pain (3), heartburn (2), and dizziness (2).

### Recommendations of Herbal Medicines

As presented in Table 7, family members, friends, or neighbors (87%) were the main recommending source, whereas only 20.9% reported that their doctor had recommended them to use HM.

**TABLE 7:** Patterns of herbal medicine use during pregnancy.

Variables	Number (%)
Recommending source (N = 177)—multiple choice	
Family/friends/neighbour	154 (87.0)
Doctor	37 (20.9)
TV/radio/internet	11 (6.2)
Newspaper/magazine	9 (5.1)
Midwife/health worker	3 (1.7)
Self	1 (0.6)
Frequency of use (N = 176)	
Daily	60 (34.1)
Occasionally	53 (30.1)
Twice or more a week	40 (22.7)
Only once	13 (7.4)
Weekly	10 (5.7)
Disclosed with doctor or midwife (N = 178)	
Yes	62 (34.8)
No	116 (65.2)
Reason of non-disclosure (N = 116)	
The doctor did not ask	60 (51.7)
It was not important	52 (44.8)
No antenatal care visit	3 (2.6)
Should have informed but forgot	1(0.9)
Reason for using herbal medicines (N = 178)—multiple choice	
I believe it is safe	99 (55.6)
I believe it is effective	84 (47.2)
Family tradition/culture	35 (19.7)
It is cheap and accessible	9 (5.1)
Unsatisfied with modern medicine	6 (3.4)
Reason for not using herbal medicines (N = 122)—multiple choice	
It is not effective	37 (30.3)
I am satisfied with modern medicine	35 (28.7)
It is not safe	23 (18.9)
It is expensive and difficult to get	18 (14.8)
My family did not let me use	12 (9.8)
My doctor/midwife did not let me use	3 (2.5)

### Frequency of Herbal Medicine Use

Among HM users (Table 7), 34.1% reported using it daily, occasionally (30.1%), twice or more a week (22.7%), only once (7.4%), and weekly (5.7%).

### Disclosure of Using Herbal Medicine

Among 178 users (Table 7), only 34.8% disclosed it to their doctor or midwife. The most frequently reported reasons for non-disclosure were doctor did not ask (51.7%), followed by it was not important to disclose (44.8%), and no antenatal visit (2.6%).

### Reasons for Using Herbal Medicine

Reasons for HM use during pregnancy are given in Table 7. Among 178 users, the most common reasons for using HM were the belief that it was safe (55.6%), was effective (47.2%), family culture/tradition (19.7%), accessible and cheap (5.1%), and dissatisfaction with modern medicine (3.4%). The most common reported reasons among 122 non-users of HM were disbelief in its effectiveness (30.3%), satisfaction with modern medicine (28.7%), disbelief in its safety (18.9%), and belief that it was inaccessible and expensive (14.8%).

## Discussion

This is the first pharmacoepidemiological study in Yemen that investigated the use of HM and its rationality during pregnancy. For this cross-sectional study data were collected through a survey of postpartum women in three hospitals in Sana’a, Yemen. The results show that several women in Yemen use HM during pregnancy.

It was found that approximately 60% of the women used at least one type of HM. This prevalence is significantly high because the majority of the sample resided in urban areas, and more than 85% of the participants resided within 1 hour of the nearest health facility. The results indicate a higher prevalence than reported in Asian and non-Asian countries (Heitmann et al., 2015; Ahmed et al., 2018). The variance in the prevalence of using HM could be due to factors such as socio-demography, culture, and uptake of health care services.

In this study, higher level of education, residence in urban areas, travel time to the nearest health facility, employment, higher number of antenatal visits, use of HM prior to last pregnancy, *Khat* (*C. edulis* C) chewing in pregnancy, exposure to secondary smoking, morbidities during pregnancy, and age at first pregnancy were predictors of using HM during pregnancy. Similar relationships have been reported previously (Mothupi, 2014; Masood and Al-Mansoob, 2015; Pallivalapila et al., 2015; Hwang et al., 2016). Women used HM mainly due to the belief that it was safe and effective, consistent with previous studies (Hwang et al., 2016; Mekuria et al., 2017; Ahmed et al., 2018). Moreover, HM was frequently used on a daily and occasional basis during the pregnancy. Similar findings were reported from other studies (Amasha and Jarrah, 2012; Hwang et al., 2016; Ahmed et al., 2018). From these results it can be deduced that HM was consumed to manage symptoms and to prepare for delivery.

Women during pregnancy were mainly recommended to use HM by family, neighbors, and friends, consistent with reports from Bangladesh and Iraq (Hwang et al., 2016; Ahmed et al., 2018). These findings make sense because friends and family are easily accessible, and often well-trusted (Ahmed et al., 2018). Moreover, most of the women did not reveal their use of HM in pregnancy to healthcare providers. The most common reason for the non-disclosure was that the “doctor did not ask”. Studies conducted in Bangladesh and Iraq support these findings (Hwang et al., 2016). This not only highlights women’s unawareness or indifference towards possible safety issues related to HM use during pregnancy but also highlights a gap in communication among doctors and patients.

Cinnamon (*C. verum* L), the most frequently consumed modality, was mainly consumed to facilitate childbirth. Consumption of Cinnamon (*C. verum* L) to facilitate delivery was previously highlighted in a study from Palestine (Jaradat and Adawi, 2013). Abortifacient and oxytocic properties of the herb have already been reported (Eid and Jaradat, 2020). While the effect of Ginger (*Z. officinale* Z) in treating nausea during pregnancy has already been recognized (Hu et al., 2022). Moreover, Barley (*H. vulgare* P) was used to treat urinary tract infections. When boiled with water, and then cooled down, Barley (*H. vulgare* P) has been traditionally consumed to mitigate symptoms of urinary tract infections, such as cystitis (Saquib Hussain et al., 2020).

As herbs contain active pharmacological ingredients and may cause biochemical changes, it is essential to evaluate the scientific evidence for their safe use during pregnancy. Clinical evidence on the safety of several herbs found in this research has not been established yet. For instance, ingestion of Cinnamon (*C. verum* L) is contraindicated during pregnancy because of possible fetal malformation (Kennedy et al., 2016; Hajimonfarednejad et al., 2019). Doses of Barley (*H. vulgare* P) greater than foods should not be ingested without the supervision of a healthcare practitioner as its safety is not studied in pregnancy (Ahmed et al., 2017). There is evidence of abortifacient, anti-fertility, and hypoglycemic activity from ingesting Fenugreek (*Trigonella foenum-graecum* F); therefore, it is not recommended during pregnancy (Kennedy et al., 2016). The use of Roselle extract (*Hibiscus sabdariffa* M) combined with an iron tablet showed a significant improvement in anemia during pregnancy (Soejoenoes and Wahyuni, 2017).

In absence of a robust spontaneous reporting system in poor countries such as Yemen, participants of this study were asked to report any adverse effects after their HM use. Among 178 users of HM, there were 15 reports of adverse effects. In this study, the use of Cinnamon (*C. verum* L) produced adverse effects of diarrhea, nausea, abdominal pain, skin rash, heartburn, burning micturition, and dizziness. Most of these adverse effects have already been highlighted (Hajimonfarednejad et al., 2019). In the literature, side effects after the use of HM during pregnancy have been reported (Jang et al., 2017).

Results of the study show a significant relationship between maternal consumption of HM and complications during labor and after delivery. No significant difference was found among users and non-users for gestational age and weight of the baby. Elsewhere, an increased incidence of threatening miscarriage and preterm labor were reported with regular use of Chamomile (*M. recutita* A) and Licorice (*G. glabra* F) (Cuzzolin et al., 2010). However, the use of these two was not reported in this study. The use of HM was significantly associated with cesarean section. However, a recent systematic review did not find such an association (Zamawe et al., 2018). Moreover, chances of experiencing prolonged labor and abnormal presentation were higher in users. With deeper analyses, it was found that users of Cinnamon (*C. verum* L) were more likely to have a cesarean section and their babies were more frequently smaller as compared to non-users. The use of Cinnamon (*C. verum* L) is contraindicated during pregnancy due to its possible relationship with a fetal malformation (Kennedy et al., 2016). In the current study, however, the number of birth defects was too small to deduce any causal relationship. Incidence of abnormal presentation and prolonged labor was also higher in regular users of Barley (*H. vulgare* P). These two conditions are complex phenomena and could be caused by several factors (Cetin et al., 2015). Therefore, it is not easy to establish the causal relationship between these and the use of HM. Nonetheless, these results indicate concerns and highlight the significance of the safe use of HM during pregnancy.

Although this research was not aimed at determining the efficacy of HM, a few interesting findings need to be mentioned. Garlic (*A. sativum* A), having polysulfides, can lower hypertension (Ried and Fakler, 2014). In this research, several women used Garlic (*A. sativum* A) and Roselle (*H. sabdariffa* M) to control hypertension during pregnancy. However, use of HM in pregnancy was significantly related with hypertension during pregnancy and after childbirth. This indicates that there was no significant decrease in the hypertension following use of Garlic (*A. sativum* A) and Roselle (*H. sabdariffa* M). Ingestion of Barley (*H. vulgare* P) was significantly related with urinary tract infections in pregnancy. This relationship was validated by other findings where women used Barley (*H. vulgare* P) to alleviate discomfort with urinary tract infections in pregnancy. Women consuming Ginger (*Z. officinale* Z) had a higher frequency of oligohydramnios. It can be explained by other results where women used Ginger (*Z. officinale* Z) to control nausea and vomiting. Vomiting may cause dehydration that may result in oligohydramnios during pregnancy (Lindower, 2017).

The current study has some limitations. The data was collected from three hospitals in Sana’a city. Therefore, the sample does not represent the whole population of Yemen and the findings cannot be generalized to the whole country. The results of the study should be interpreted with caution because it presents the only association between the HM use and the pregnancy outcomes, not the cause and effect. Moreover, this research could not determine the dose, frequency, and duration of using individual HM. There is also a chance of recall bias in the survey. However, an interview just before the discharge could lower this risk. Furthermore, potential mechanisms of toxicities of the selected HM are not provided. It can be considered in future studies. The major strength of the study is that it is the first attempt to see whether it is rational to use HM during pregnancy in Yemen.

## Conclusion

The popularity of HM use during pregnancy in Yemen usually without consulting a healthcare practitioner is alarming. Rational use of HM can be an effective way to manage symptoms during pregnancy in regions already depleted in resources and hit with a crisis. In contrast, the results of the current study show that the choice of HM for effectiveness and safety is often based on recommendations of family and friends instead of clinical evidence. This highlights the need to evaluate the safety and effectiveness of HM *via* pharmacoepidemiological research to generate scientific evidence. Then, there is a need to conduct mass awareness campaigns and programs to facilitate the appropriate use of HM that is based on clinical evidence Moreover, there is a need to develop guidelines in clinical settings for healthcare providers to facilitate provider-patient communication to ensure rational use of HM.

## Data Availability

The raw data supporting the conclusion of this article will be made available by the authors, without undue reservation.

## Abbreviations

HM, herbal medicine; WHO, World Health Organization; EBF, exclusive breastfeeding.
